# Evaluating the Impact of Sex-Biased Genetic Admixture in the Americas through the Analysis of Haplotype Data

**DOI:** 10.3390/genes12101580

**Published:** 2021-10-07

**Authors:** Linda Ongaro, Ludovica Molinaro, Rodrigo Flores, Davide Marnetto, Marco R. Capodiferro, Marta E. Alarcón-Riquelme, Andrés Moreno-Estrada, Nedio Mabunda, Mario Ventura, Kristiina Tambets, Alessandro Achilli, Cristian Capelli, Mait Metspalu, Luca Pagani, Francesco Montinaro

**Affiliations:** 1 Estonian Biocentre, Institute of Genomics, University of Tartu, Riia 23b, 51010 Tartu, Estonia; ludovica.molinaro@ut.ee (L.M.); rodrigo.flores@ut.ee (R.F.); davide.marnetto@ut.ee (D.M.); kristiina.tambets@ut.ee (K.T.); mait.metspalu@ut.ee (M.M.); lp.lucapagani@gmail.com (L.P.); francesco.montinaro@gmail.com (F.M.); 2 Department of Biology and Biotechnology “L. Spallanzani”, University of Pavia, 27100 Pavia, Italy; marcorosario.capodiferro01@universitadipavia.it (M.R.C.); alessandro.achilli@unipv.it (A.A.); 3 Department of Medical Genomics, GENYO, Centro Pfizer—Universidad de Granada—Junta de Andalucía de Genómica e Investigación Oncológica, Av de la Ilustración 114, Parque Tecnológico de la Salud (PTS), 18016 Granada, Spain; marta.alarcon@genyo.es; 4 National Laboratory of Genomics for Biodiversity (LANGEBIO), CINVESTAV, Irapuato, Guanajuato 36821, Mexico; andres.moreno@cinvestav.mx; 5 Instituto Nacional de Saúde, Distrito de Marracuene, Estrada Nacional N°1, Província de Maputo, Maputo 1120, Mozambique; nediojonas@gmail.com; 6 Department of Biology-Genetics, University of Bari, 70126 Bari, Italy; mario.ventura@uniba.it; 7 Department of Zoology, University of Oxford, Oxford OX1 3SZ, UK; cristian.capelli@unipr.it; 8 Department of Chemistry, Life Sciences and Environmental Sustainability, University of Parma, 43124 Parma, Italy; 9 Department of Biology, University of Padua, 35131 Padua, Italy

**Keywords:** admixture, American populations, sex-biased imbalance, haplotypes, human migrations

## Abstract

A general imbalance in the proportion of disembarked males and females in the Americas has been documented during the Trans-Atlantic Slave Trade and the Colonial Era and, although less prominent, more recently. This imbalance may have left a signature on the genomes of modern-day populations characterised by high levels of admixture. The analysis of the uniparental systems and the evaluation of continental proportion ratio of autosomal and X chromosomes revealed a general sex imbalance towards males for European and females for African and Indigenous American ancestries. However, the consistency and degree of this imbalance are variable, suggesting that other factors, such as cultural and social practices, may have played a role in shaping it. Moreover, very few investigations have evaluated the sex imbalance using haplotype data, containing more critical information than genotypes. Here, we analysed genome-wide data for more than 5000 admixed American individuals to assess the presence, direction and magnitude of sex-biased admixture in the Americas. For this purpose, we applied two haplotype-based approaches, ELAI and NNLS, and we compared them with a genotype-based method, ADMIXTURE. In doing so, besides a general agreement between methods, we unravelled that the post-colonial admixture dynamics show higher complexity than previously described.

## 1. Introduction

Present-day populations living in the Americas trace their ancestry to Indigenous groups, together with influxes from multiple sources from Africa and Eurasia, due to a complex history of admixture following the Atlantic Slave Trade, the Colonial Era, and more recent migration phenomena [1]. In the last decade, a substantial number of genomic surveys of American populations highlighted the extreme heterogeneity in the continents, also reflecting the diverse demographic history of different areas [2,3,4,5,6,7].

A general imbalance in the proportion of disembarked males and females has been documented during the Slave Trade and the colonial Era and, although less prominent, in more recent times [8,9,10]. In fact, early surveys based on uniparental markers suggested the existence of a sex-biased admixture history, with a higher degree of American and African mitochondrial DNA when compared to their Y-chromosome counterpart [11,12,13,14,15]. This evidence is possibly the result of more European males mating with American and African females. 

The advent of genomic data allowed the exploration of sex-biased admixture dynamics by comparing ancestry proportions estimated in autosomal and X chromosomes [4,16,17,18,19]. In this scenario, if the number of mating males and females from a given ancestry is similar, its inferred proportion on X and autosomal chromosomes should not be significantly different. On the other hand, an excess of the expected ancestral proportion for the X or autosomal chromosomes would be compatible with a higher number of mating females or males, respectively [20]. In this context, the ancestry inference has usually been estimated using STRUCTURE-like methods, which, harnessing different statistical frameworks, assign the individual genomic data to an arbitrary number of “ancestries”. Using this or similarly related techniques [6,21], a widespread imbalance in ancestry proportions for many American populations has been observed, characterised by a variable degree of X/autosomal imbalance. In Ongaro et al. 2019 [6], where we applied ADMIXTURE [22], we found that the distribution of autosomal vs. X chromosomes for the European ancestry is significantly higher in all comparisons, suggesting a more significant contribution of European males than females in the gene pool of American populations. As for the Indigenous American ancestry, we estimated a higher proportion of chrX than the autosomes in all populations. In contrast, when considering African ancestry, many populations do not show any signs of sex imbalance. Indeed, in only eight out of 19 comparisons, the autosomal proportion was significantly lower than that inferred from the X chromosome. These results are in contrast with historical records documenting a higher number of disembarked male slaves from Africa [23] and might reflect complex admixture dynamics. They could also reflect limitations in the approach exploited in Ongaro et al. 2019 [6], as previously suggested [24].

Although haplotype-based methods have been successfully harnessed to uncover the genetic structure of worldwide human populations and their determinants [25,26,27], to our knowledge, they have not been used to explore the extent of the sex bias admixture dynamics in the Americas.

In this study, we analysed genome-wide data for more than 5000 individuals from the Americas to assess the presence, direction and magnitude of sex-biased gene flow in the Americas. In doing so, besides a general agreement with genotype-based methods, we unravelled a higher than previously described complexity in the post-colonial admixture dynamics.

## 2. Materials and Methods

### 2.1. Autosomal and X Chromosome Datasets

Both the analysed genome-wide datasets, autosomal and X chromosome, were recovered from Ongaro et al. 2019 [6]. The former was filtered using PLINK ver. 1.9 (city, country) [28] to include only SNPs and individuals with a genotyping success rate >97%, retaining a total of 251,548 autosomal markers. As for the latter, we intersected SNPs from the chrX that were present in both our main datasets and in the 1000 Genomes Project samples [29]. For this study, we used the reference genome version b37.

We revised and imputed sex assignments based on X chromosome data using the --impute-sex command in PLINK. A male or female call is made when the rate of homozygosity is >80% and <20%, respectively. Individuals for which sex imputation was ambiguous were removed, and heterozygous SNPs in male X chromosomes were set as missing. After this step, only samples and positions with a genotyping rate >97% were retained: 5227 SNPs in 9811 individuals, of which 5418 belonged to 17 admixed American populations, while 4393 to populations from all other the world. The same set of individuals was extracted from the filtered autosomal dataset with 258,720 SNPs.

### 2.2. Admixture (ADM)

As in Ongaro et al. 2019 [6], we performed LD pruning (--indep-pairwise 200 50 0.2) in both X chromosome and autosomal datasets, resulting in a total of 2519 and 116,912 SNPs, respectively. We ran separate unsupervised ADMIXTURE (version 1.3.0(city, country) [22]) analyses for the two datasets using *K* values = 3 and 10 independent runs. We used the option “--haploid = ‘male:23” to properly treat male individuals and chose the best run according to the highest log-likelihood value. Finally, we performed paired Wilcoxon tests to test for significant differences between the ancestry proportions observed in the autosomes versus the X chromosome and used Bonferroni correction for multiple-testing (adjusted *p*-value < 0.05). We evaluated similarities in the autosomal/X chromosome ratio distribution by applying a Wilcoxon distribution. 

### 2.3. Phasing

To proceed with the haplotype-based methods, we reconstructed the maternal and paternal gametic phase. We used the Segmented Haplotype Estimation and Imputation tool (ShapeIT2) software (city, country) [30], using the HapMap37 (city, country) human genome build 37 recombination map. We phased the chromosome X dataset adding the option --chrX.

### 2.4. Local Ancestry Inference with ELAI

We estimated the local ancestry for genomic fragments of the target American individuals with ELAI software(city, country) [31], using the following reference populations: Yoruba (YRI) and Mozambique for Africa, Chinese Han (CHB) and Japanese (JPT) for Asia, Spanish (IBS), British (GBR) and Tuscany (TSI) for Europe and Wichi and Karitiana for Indigenous American ancestry (Supplementary Table S1).

We ran ELAI on phased data, using the following parameters: -C 4, to infer a four-way admixture; -c 20 for twenty lower-layer clusters; -mg 12 to indicate twelve generations since the admixture and -s 20 for twenty Expectation Maximisations (EM) iterations, as recommended in ELAI manual [31]. 

We ran ELAI on each autosomal chromosome and X chromosome; we performed ten independent runs and averaged the results for each analysis.

### 2.5. Non-Negative Least Square Haplotype-Based Ancestry Estimation

We used the copying vectors obtained with CHROMOPAINTER [32] described in Ongaro et al. 2019 [6]. We identified the most closely ancestrally related donor population for each admixed population by comparing their copying vectors to copying vectors inferred in the same way for each of the donor clusters using a slight modification of the non-negative least square (NNLS) function in R 3.5.1 [33], and following the approach reported in previous surveys [2,6,34]. In detail, for each recipient population and each individual, we decomposed the ancestry of that group as a mixture (with proportions summing to 1) of each sampled potential donor cluster by comparing the “copying vector” of donor and recipient populations and individuals. Then, we grouped the results based on continental variations based on the fineSTRUCTURE clusters obtained in Ongaro et al. 2019 [6], and we report the results in Supplementary Table S2A.

### 2.6. Bayesian Haplotype-Based Ancestry Estimation (SOURCEFIND)

We applied SOURCEFIND [5] to estimate the ancestral composition of recipient individuals both on the chromosome X and the autosomes. We modelled the copying vector of each admixed individual obtained with CHROMOPAINTER as a weighted mixture of copying vectors from the donors. We used as parameters: self.copy.ind = 0, number of total (num.surrogates) and expected (exp.num.surrogates) surrogates equal to 8 and 4, respectively; performing (total number of MCMC iterations) 200,000 iterations thinned every 1000, and preceded by a burn in step of 50,000. Furthermore, we assigned equally-sized proportions to the surrogates (num.slots = 100). For each recipient individual, we combined 10 independent runs extracting and averaging the estimates with the highest posterior probability, weighted by their posterior probability. The results are summarised in Supplementary Figure S1. However, we will not discuss these results because the chromosome X one is not very reliable, probably because of the low number of SNPs left after filtering.

### 2.7. Comparison between Different Methods

We tested the correlation between results coming from different methods. At first, we calculated the correlation between the continental individual proportion of chrX obtained with ADMIXTURE and one of the other methods on a population level (ADMIXTURE vs. ELAI, ADMIXTURE vs. NNLS). Then, we repeated the same for the results regarding the autosomal proportions. The results are summarised in Supplementary Table S2B,C.

To calculate the correlation coefficient, we ran two tests with R:

- cor.test, using the Pearson method, obtaining the rho coefficient.
- lm, to fit the linear model, obtaining the adjusted r squared.

The supplementary tables reported only the correlation coefficient obtained from the Pearson method because the results were very similar to the linear model.

Then, to understand if the differences between the various methods were statistically significant, we performed a paired Wilcoxon test with R.

### 2.8. Sex-Biased Imbalance with All Methods

For each population and method, we investigated the differences between the individual’s autosomes’ continental ancestry proportions versus the ones of chrX. To be consistent in our analyses, we performed two filtering steps: first, we filtered out those individuals with less than 25% of a specific ancestry in both autosomes and chrX and then we kept only those populations with 15 individuals or more. In this way, we obtained a dataset containing 5242 admixed American individuals (Supplementary Table S1). Finally, we performed paired Wilcoxon tests on R on all the possible combinations (Supplementary Table S2D). 

### 2.9. Ratios 

We calculated the ratios between the autosomal and the chrX continental proportions of the individuals (Supplementary Table S2F–H). Then, we compared the resulting ratios from the different methods using the Wilcoxon test and the cor.test (Pearson method) in R. The results are summarised in Supplementary Table S2E.

## 3. Results

### 3.1. Comparison between Haplotype-Based and ADMIXTURE Estimates

In this study, we applied two haplotype-based methods (ELAI and NNLS) on a dataset composed of 5242 individuals for the X chromosome and the autosomes (Supplementary Table S1). Our purpose was to compare the distribution of continental ancestry proportion in seventeen American populations (Supplementary Table S1A) applying three different methods. 

**Figure 1:**
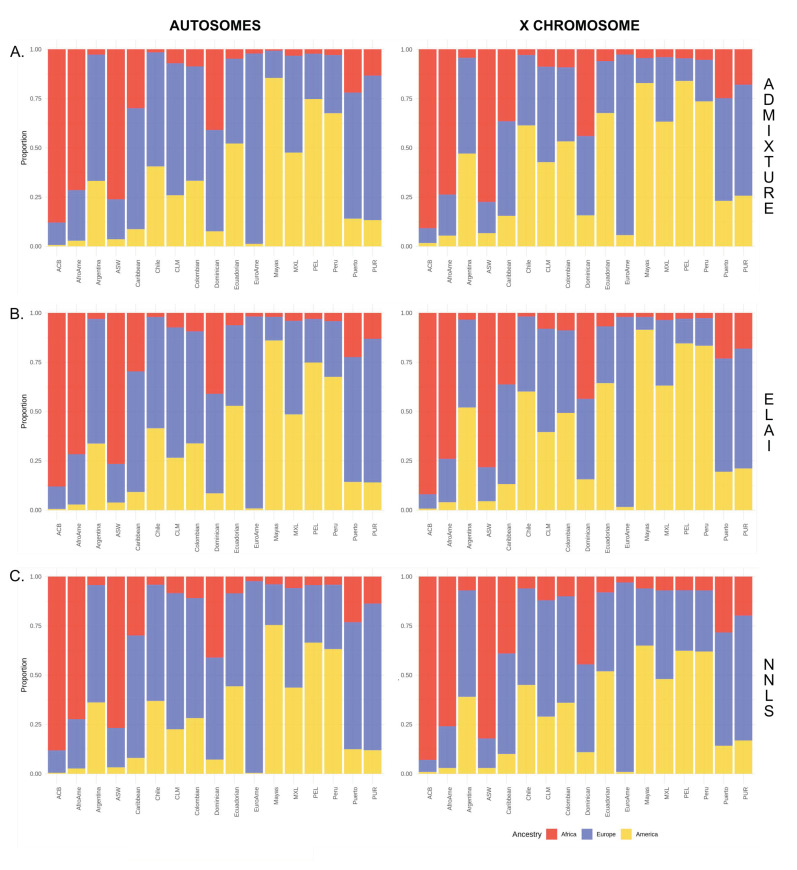
Overview of autosomal and chromosome X global continental proportions in admixed American populations inferred with three different methods: (**A**) ADMIXTURE, (**B**) ELAI and (**C**) NNLS.

First, we assessed the global reliability for autosomal and X chromosome of ELAI [31], a recently developed and efficient local ancestry estimation method, and NNLS, which exploits haplotypic “copying vectors” obtained by CHROMOPAINTER [32] to get the global ancestry estimation, against ADMIXTURE [22], which uses genotype data. The autosomal and chromosome X global continental proportions obtained with the three methods are shown in Figure 1.

Both the two haplotype-based methods displayed a very high correlation with ADMIXTURE component proportions for autosomal and X chromosomes, with most of the comparisons having an *R^2^* > 0.9 (Supplementary Table S2B,C). For African ancestry in autosomes, the R^2^ is always higher than 0.9, except for Argentina (0.69), Chile (0.43) and MXL (0.82) when we correlate ADM and NNLS. As for the chrX, the R^2^ between ADM and ELAI is always higher than 0.9, except for the American ancestry in ACB (0.78) and EuroAme (0.79) and the African ancestry in Chile (0.72). Moreover, the R^2^ between ADM and NNLS is higher than 0.9 in the majority of cases, except for the European ancestry of Chile (0.83), EuroAme (0.84) and Peru (0.88), the American ancestry of ACB (0.68) and EuroAme (0.77), and finally the African ancestry of Argentina (0.82), Chile (0.18), EuroAme (0.86) and MXL (0.7). The low correlation coefficients are probably related to the small sample size of some populations under analysis and their extreme ancestral proportions, as it is for Chile (only 22 individuals and very low African ancestry, less than 10% on average, Supplementary Table S2A). 

When the individual ancestry proportions were further investigated, we registered a very subtle difference between ELAI and ADMIXTURE, with the absolute difference between estimates ranging between 0.25% (American ancestry of ACB) and 1.5% (African ancestry of Ecuadorian) for the autosomes and between 0.8% (African ancestry of PEL) and 5.6% (European ancestry of EuroAme) for the chrX (Supplementary Table S2B). Nevertheless, for European ancestry, we found 11 and 12 significant comparisons in autosomal (comparison showing a lower European ELAI estimate *p*-value) and the chrX (comparison showing a higher European ELAI estimate *p*-value), with opposite directions, utilising paired Wilcoxon tests (Supplementary Table S2B).

When we looked at the absolute difference within estimates between NNLS and ADMIXTURE, we found higher differences than in the previous comparisons (Supplementary Table S2C). The autosomes range from 0.4% (American ancestry of ACB) to 11.74% (American ancestry of Mayas). In comparison, the chrX range from 1.2% (American ancestry of ACB) to 23% (American ancestry of Mayas).

In details, for the autosomes, the inferred European proportions for NNLS were significantly higher than the ADMIXTURE ones for eight populations (Argentina, Chile, CLM and Colombian from Colombia, Ecuadorian, MXL from Mexico, PEL and Peru from Peru) and lower for five groups (ACB, AfroAme, ASW from the US, Caribbean and EuroAme). In contrast, we observed consistent results for chrX, for which nine populations showed higher European ancestry for the NNLS (Argentina, Chile, CLM, Colombian, Ecuadorian, EuroAme, MXL, PEL and Peru) as reported in Supplementary Table S2C.

As for the American ancestry, we found seven populations (Argentina, Caribbean, Chile, CLM, Dominican, MXL and PUR) with higher autosomal proportions with ELAI and only one, EuroAme, with ADM; then, we obtained lower chrX proportions in 15 populations (all except Dominican and Mayas) with ELAI compared to ADM (Supplementary Table S2B). When we applied the NNLS method, we found 14 (all except ACB, ASW and Mayas) and 15 (all except ACB and Mayas) populations with, respectively, lower autosomal and chrX American proportions with NNLS compared to ADM estimates (Supplementary Table S2C).

Finally, we analysed the African ancestry. We found higher autosomal proportions of African ancestry in eight populations (AfroAme, Argentina, Chile, Colombian, Ecuadorian, MXL, PEL and Peru) with ELAI and three populations (Caribbean, EuroAme and PUR) with ADM. In contrast, we found only three populations (ACB, AfroAme and ASW) with higher chrX proportions with ELAI and another three (Argentina, Chile and EuroAme) with ADM (Supplementary Table S2B). As for the second haplotype-based method applied, the African proportions obtained from the NNLS are always higher than those obtained from ADM, except for ACB when the autosomal proportions are evaluated and Mayas for both chrX and autosomes (they are not significant after Bonferroni correction).

### 3.2. Inference of the Sex-Biased Imbalance through Different Methodologies

**Figure 2:**
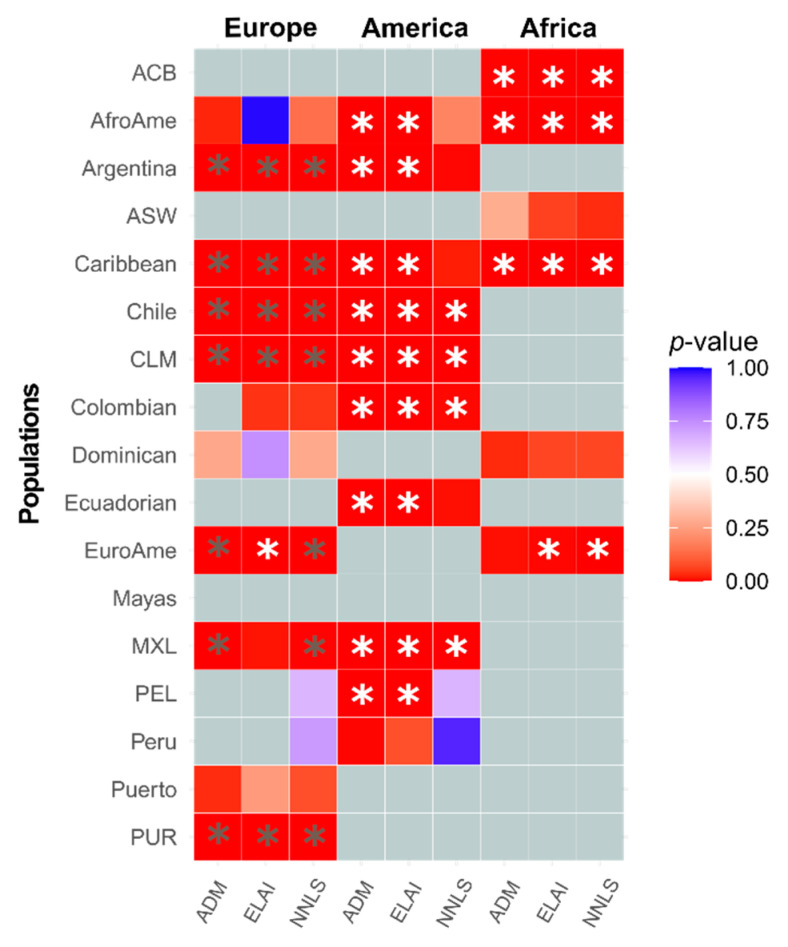
Significance of autosomes vs. X continental proportions in admixed American populations. Squares are coloured based on the adjusted *p*-value from the Wilcoxon tests reported in Supplementary Table S1D. Grey squares represent those cases in which the population did not pass the filter (average ancestry proportion > 25% and more than 15 individuals). Square colors are based on the *p*-value as shown in the legend. Grey asterisk indicates that the proportion of the autosomes is significantly higher than those of chromosome X after Bonferroni correction; white asterisk indicates that the proportion of chrX is significantly higher than the autosomal one after Bonferroni correction.

We investigated the differences between the autosomal ancestry proportions and the chrX at the continental level for each population and each method employed. First, for each method, we filtered out those individuals with less than 25% of a specific ancestry in both autosomes and chrX. Then, we retained only those populations with at least 15 individuals. We performed paired Wilcoxon tests on all the possible combinations. The results are listed in Supplementary Table S2D and are also presented in Figure 2. 

We observed a general excess of European ancestry in the autosomal genome compared with chrX, consistent with a stronger male ancestral contribution. Six populations showed a consistent result regardless of the method employed: Argentina, Caribbean, Chile, CLM, PUR and MXL (although the latter was not significant after Bonferroni correction in ELAI). EuroAme showed the same signal only with ADM and NNLS, but opposite direction with ELAI, suggesting that this method might not recover with high confidence marginal secondary ancestries, possibly because of the low numbers of markers characterising the chrX dataset (5227 SNPs). In fact, for all the three analyses, European Americans have on average, less than 5% of non-European ancestry (Supplementary Table S2A). When considering the American ancestry, we observed the same pattern in all the three methodologies applied (Figure 2). 

In detail, seven populations showed significantly higher American proportions for chrX than autosomes, a signal consistent with a higher female ancestry contribution (Chile, CLM, Colombian and MXL after Bonferroni in all methods, while Argentina, Caribbean and Ecuadorian do not maintain the significance after the Bonferroni correction when NNLS is applied). Moreover, this pattern is also detected in two additional populations (AfroAme and PEL) when ADM and ELAI are employed. It is also interesting to note that for the two Peruvian populations under study (PEL and Peru), we observed the opposite pattern (autosomes higher than chrX), especially for Peru (*p*-value = 0.05). These results suggest that the NNLS method assigns less windows of the American ancestry in the chrX dataset compared to the autosomal one, probably because of the SNPs low density which cannot discern enough American ancestry compared to others.

Then, when we considered African ancestry, we found a high consistency in the results with four populations (ACB, AfroAme, Caribbean and EuroAme; the latter is not significant after Bonferroni correction in ADM), showing a statistically significantly higher proportion of chrX than autosomes, again suggesting a higher contribution from female ancestors.

### 3.3. Comparison of the Autosomal/chrX Ratios between Methods

To further evaluate the impact of the sex-biased admixture in the Americas, we assessed the Autosomal/X imbalance ratio differences among the three methodological approaches adopted (Supplementary Table S1E). In doing so, we kept only those populations present in each method’s pair evaluated. 

**Figure 3:**
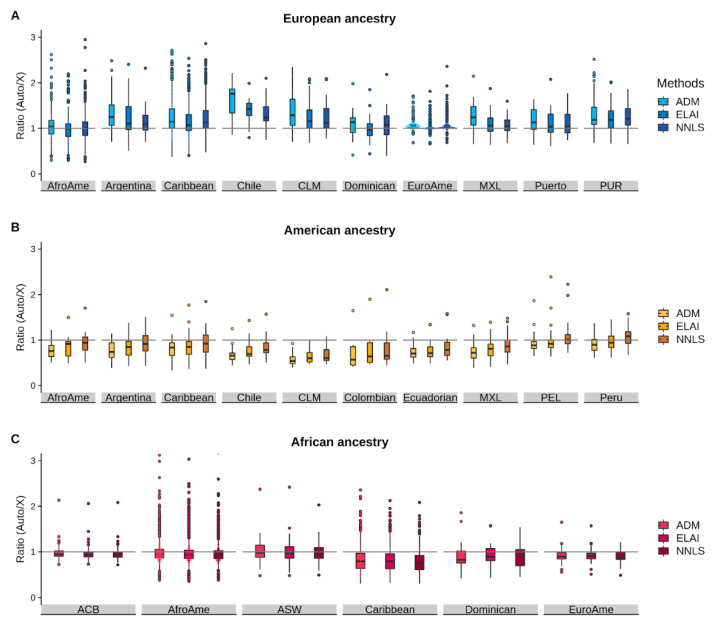
Ratios of autosomal vs. chromosome X continental proportions in admixed American populations. Each boxplot shows the ratio of autosomal to chrX ancestry proportion for (**A**) European, (**B**) Indigenous American, and (**C**) African continental components as inferred by ADMIXTURE, ELAI and NNLS in admixed American populations. Boxplots refer to the interquartile range, and whiskers refer to data points not exceeding the interquartile range more than 1.5 times. All the other data points are considered outliers and shown as dots. This Figure was realised using the library ggplot2 of R. These results are also reported in Supplementary Table S1E–H.

First, we analysed the resulting ratios (autosomes proportions divided by the chrX proportions) for the European ancestry (Figure 3A). In most populations, the median ratio is higher than 1, suggesting a sex imbalance towards the autosomes. The median values are less than 1 in AfroAme (Min = 0.3, Max = 2.2), Dominican (Min = 0.4, Max = 1.9) and EuroAme (Min = 0.7, Max = 1.8) when ELAI is applied. 

When we compared the haplotype-based methods with ADMIXTURE, we found higher ratios with ADM than ELAI in all the populations under analysis (Dominican is not significant after Bonferroni correction), suggesting that the estimated European autosomal proportions are lower when ELAI is applied. The same pattern was obtained from the comparison of NNLS with ELAI, where we observed that in seven populations (AfroAme, Argentina, Caribbean, Chile, CLM, EuroAme and MXL) out of 10, the estimated European ancestry ratios are higher when ADM is applied than NNLS.

These results are also evident in Figure 3, wherein most of the population’s median value of the ADMIXTURE results is higher than ELAI and NNLS. 

As for the American ancestry (Figure 3B), in all the populations, the median ratio is lower than 1, suggesting a higher admixture contribution from American females (chrX). The only exceptions are obtained with NNLS in PEL and Peru, in which the median ratio is 1 (Min = 0.7, Max = 2.2) and 1.1 (Min = 0.7, Max = 1.6), respectively. When we performed the Wilcoxon test to evaluate the differences between the methodologies applied, we obtained a statistically significant difference for all the populations under analysis (except AfroAme), with higher autosomal proportions obtained with ELAI. Therefore, with ADM, we obtained higher chrX proportions. Moreover, in seven out of ten populations (AfroAme, Argentina, Caribbean, Chile, MXL, PEL and Peru), the ratios are higher for NNLS when compared with ADMIXTURE. As already mentioned above, these results might suggest that the NNLS method assigns less genomic segments of the American ancestry in the chrX dataset compared to the autosomal one. This idea is also confirmed by the fact that when we compared ELAI with NNLS, in all populations (AfroAme, Argentina, Caribbean, Chile, CLM, Ecuadorian, MXL, PEL and Peru) except for Colombian, the estimated American proportions of the autosomes are higher with the NNLS than ELAI. This pattern is also easily visible in Figure 3B, where the median of the NNLS boxplots is always higher than the other two.

Lastly, we analysed the sex imbalance related to African ancestry. The median value of the ratio (Autosomes/chrX) is always lower than 1, suggesting a higher impact in the admixture events of chrX. However, we found only one population (AfroAme) for the African ancestry with a statistically significant difference, with higher autosomal proportions estimated with ADM. We observed a similar pattern in all analysed populations (ACB, AfroAme, ASW, Caribbean and EuroAme), except for Dominican, when comparing ADM and NNLS, with NNLS values of chrX consistently lower. Interestingly, the African ancestry represents the one with fewer differences, as also shown in Figure 3C. Additionally, Caribbean populations differ from the others, dispensing a lower ratio in all the methods employed. However, to address these results more accurately, we would need more individuals from different populations and geographical areas. Unfortunately, many populations present in our initial dataset were filtered out because of low African proportions (Supplementary Table S2A).

## 4. Discussion

In this work, we evaluated two different commonly used haplotype-based methods, ELAI and NNLS, to assess the extent and magnitude of sex-biased admixture dynamics in ~5000 individuals from ten American countries. To our knowledge, this is the first time that such methodologies are harnessed for this purpose. 

Our results confirm a high correlation between haplotype- and genotype-based methods. However, caution should be used when NNLS is harnessed for extremely low ancestry proportions and a low number of SNPs. In fact, NNLS probably assigns less genomic segments of the American ancestry in the chrX dataset compared to the autosomal one. In addition, when we applied SOURCEFIND, a recently developed Bayesian method, we found very low proportions of the American ancestry when analysing the X chromosome data compared to the autosomes and the other methods. The reason for this poor performance is probably correlated to the low number of chrX SNPs available. Therefore, the possibility of having more SNPs and testing better priors in a future study could improve the method performance.

When the sex-biased admixture was evaluated, we found a general agreement among the three tested methods (ADMIXTURE, ELAI and NNLS). In detail, our analysis shows the signature of a higher number of mating European males rather than females for five out of ten populations. This is in line with historical records that report a strong male bias in migration at the beginning of the colonial settlements and previous genetic analysis, exploiting both uniparental systems and using genomic data [12,13,14,24,35]. 

Moreover, the evaluation of individual’s autosomes/X ratio suggests that the sex bias has been more pronounced for continental populations, possibly because of the more extended exploitation and for the occurrence of more recent labour-related migrations from Europe towards the two continents.

The opposite pattern was observed for the American ancestry, which suggests a higher number of mating females of American descent throughout the continents (AfroAme, Argentina, Caribbean, Chile, CLM, Colombian, Ecuadorian, MXL and PEL), corroborating previous surveys [3,19]. This observed imbalance is more severe in Chile, Colombia (CLM and Colombian) and Ecuador, and milder in Peru (PEL and Peru) and Mexico (MXL). This observation is consistent with previous surveys of the Indigenous American ancestry in the continents that have shown a global softer reduction in effective population size in the two countries concerning other American populations, possibly as the result of admixture between different autochthonous groups. 

Similarly, for the African ancestry, we observed a general under-representation in the autosomes for four out of six populations considered (ACB, AfroAme, Caribbean and EuroAme), reflecting the higher number of mating females compared to males, as also observed in previous surveys [4,19,21]. The absolute magnitude of the Autosomal/X ratio imbalance is lower than those observed for European and American ancestries. 

Overall, our results show that ancestry reconstruction haplotype-based methods represent a valuable tool for evaluating the sex-biased admixture and should be used to validate commonly harnessed genotype-based approaches. In addition, we confirmed the presence of a general imbalance between autosomal and X chromosome ancestry estimates, suggesting a higher number of European mating males and American or African females contributing to the genetic variation in the continents. 

Although we analysed a substantial number of individuals (>5000) from many (ten) different American countries, it is possible that more heterogeneity would emerge with the analysis of other groups, also belonging to different regions of the same countries. Therefore, studying a much higher number of individuals with a larger geographic distribution will help elucidate the phenomenon’s dynamics at a finer scale. In addition, the recent advancement offered by the development of forward in time simulations approach that allows for the modelling of very complex scenarios [36,37] is needed to understand how demographic parameters, such as time of admixture episodes and length, number of continental waves, ancestry-based assortative mating, may affect the autosomal/X ancestral estimates [38,39]. Moreover, multinomial regression approaches could provide further insights in the analysis of the sex-biased admixture history in the Americas.

## Supplementary Materials

The following are available online at https://www.mdpi.com/article/10.3390/genes12101580/s1, Supplementary Figure S1: SOURCEFIND results, Table S1: Details of the genotype data used in this study, Table S2: Summary of the sex-biased imbalance inferred using three methods.
