# Supplementary figures and images for: Evaluating the Impact of Sex-Biased Genetic Admixture in the Americas through the Analysis of Haplotype Data
Source: Genes (Basel). 2021 Oct 7;12(10):1580. doi: 10.3390/genes12101580 (PMC8535939; doi:10.3390/genes12101580)

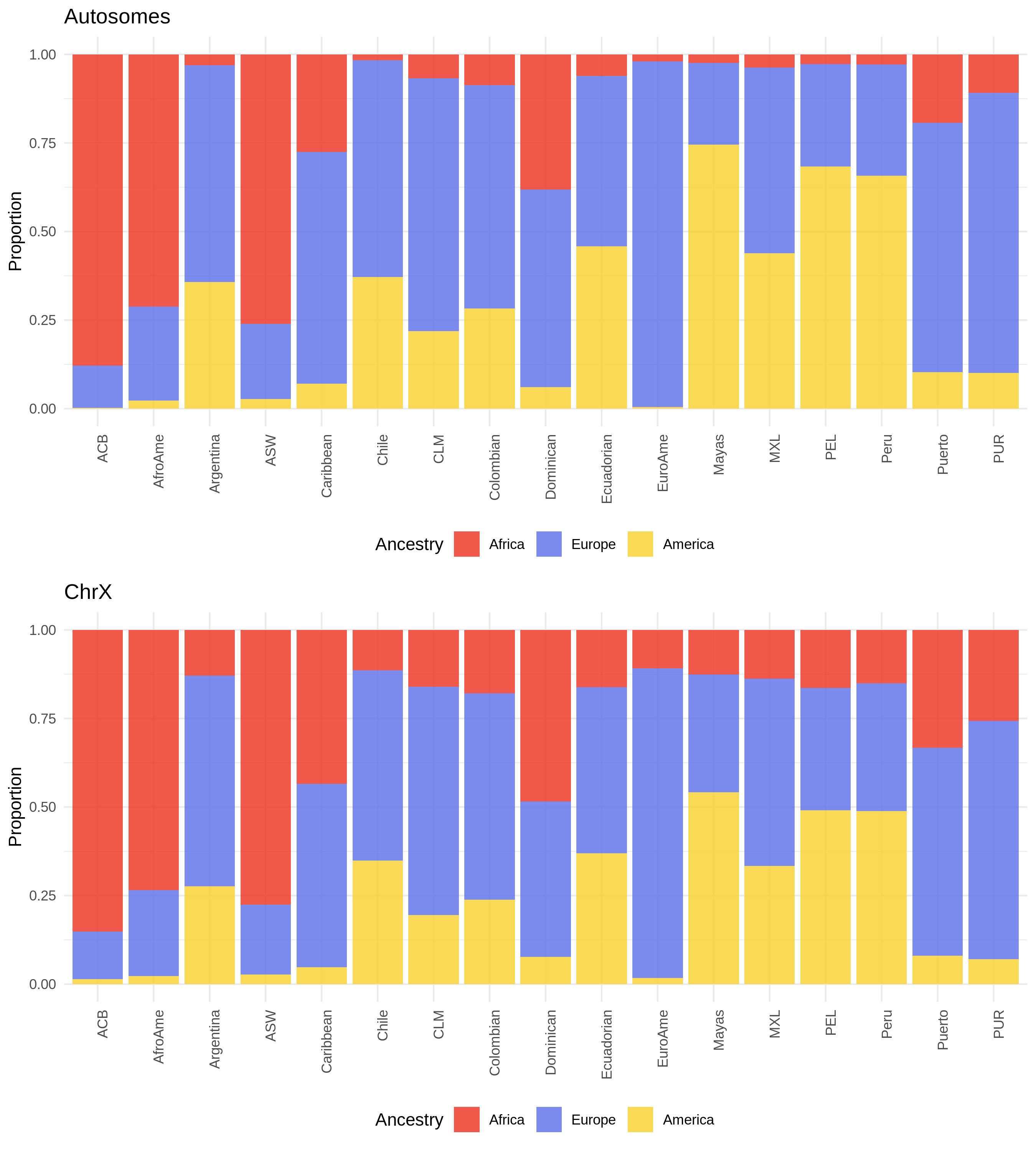

Supplement: Supplementary file 1 [file genes-12-01580-s001.zip › Supplementary/SupplementaryFigure1.png]
